# Investigation of antioxidative effects of a cardioprotective solution in heart tissue

**DOI:** 10.1007/s11010-019-03591-y

**Published:** 2019-07-24

**Authors:** Miriam Russ, Susanne Jauk, Reinhold Wintersteiger, Michaela Andrä, Iva Brcic, Astrid Ortner

**Affiliations:** 1 Department of Pharmaceutical Chemistry, Institute of Pharmaceutical Sciences, University of Graz, Schubertstraße 1, 8010 Graz, Austria; 2 Division of Transplant Surgery, Medical University of Graz, Auenbruggerplatz 29, 8036 Graz, Austria; 3 Diagnostic & Research Institute of Pathology, Medical University of Graz, Neue Stiftingtalstraße 6, 8010 Graz, Austria

**Keywords:** Malondialdehyde, Carbonyl proteins, Alpha-ketoglutarate (α-KG), HPLC/UV–Vis, Ischemia reperfusion, Oxidative stress

## Abstract

A multi-component solution, containing α-ketoglutaric acid (α-KG), 5-hydroxymethylfurfural (5-HMF), *N*-acetyl-seleno-l-methionine (NASeLM), and *N*-acetyl-l-methionine (NALM) as active ingredients, has been tested considering its supposed antioxidative effect with respect to heart transplantations. Oxidative stress was induced on isolated rat hearts through occlusion of a coronary artery and in chicken heart tissue through hydrogen peroxide. Both heart types were analyzed and the oxidative stress markers malondialdehyde (MDA) and carbonyl proteins (CPs) were determined via HPLC/UV–Vis. In both approaches, it was found that treatment with the multi-component solution led to a lower amount of MDA and CPs compared to a negative control treated with Krebs–Ringer solution (KRS). Further investigation on chicken heart tissue identified α-KG as antioxidative component in these experiments. However, numerous factors like arrhythmia, vessel dilatation, and minimization of oxidative stress effects play an important role for successful transplantation. Therefore, the investigated multi-component solution might be a novel approach against oxidative stress situations, for example at ischemia reperfusion injury during heart transplantations.

## Introduction

Graft failure is a severe problem after heart transplantations. It is caused due to the excessive formation of reactive oxygen and nitrogen species (ROS/RNS) during the peri-surgical process including explantation, transport, and implantation.

ROS generation occurs mainly at reperfusion when molecular oxygen is reintroduced into the ischemic area. Furthermore, ROS can react with nitric oxide (NO) and thereby form reactive nitrogen species (RNS)—for example peroxynitrite [1, 2]. ROS and RNS are able to cause genotoxic damage, cell dysfunction, and cell death or inactivation of enzymes by oxidizing structures such as proteins, lipids, and DNA, resulting in oxidative tissue damage [3, 4]. Overall, these effects can lead to a functional disorder of the transplanted organ in the body and result in graft failure [1]. Therefore, it is important in transplant surgery to reduce or block the effect of ROS and RNS and their following ischemia reperfusion injury to avoid this malfunction.

A novel approach to overcome this problem is a multi-component solution in combination with angiotensin-(1-7). The active ingredients of the multi-component solution are α-ketoglutaric acid (α-KG), 5-hydroxymethylfurfural (5-HMF), *N*-acetyl-seleno-l-methionine (NASeLM), and *N*-acetyl-l-methionine (NALM). α-KG and 5-HMF are described as directly antioxidative agents under different conditions [5–8]. NALM is a source of methionine, while NASeLM is a source of selenium. Both methionine and selenium are contributors of the body’s antioxidative defense system via regulation of antioxidative enzymes [9–12].

The combination showed already promising effects on the hearts of male Wistar rats at the Langendorff device considering arrhythmia, coronary flow, etc. during ischemia [13]. Angiotensin 1-7 has already been studied and proven to have antiarrhythmic and cardioprotective effects [14–16].

It was hypothesized that the multi-component solution exhibits significant antioxidative power in heart tissue, which is considered to be effective against ROS and RNS, thereby minimizing ischemia reperfusion injury. Hence, the aim of this study was to investigate the supposed antioxidative effect of the multi-component solution via the oxidative stress markers malondialdehyde (MDA) and carbonyl proteins (CPs). MDA as result of lipid peroxidation and CPs as result of protein oxidation are both stable products and reflect the local occurrence of ROS. They are widely applied markers for the determination of oxidative stress [17].

Simulation of oxidative stress was carried out by applying two different approaches: via myocardial infarction on the hearts of male Wistar rats and via oxidation of chicken heart tissue through hydrogen peroxide. Krebs–Ringer solution was used as negative control during experiments. MDA and CPs were quantified in the heart tissue with HPLC according to a formerly developed method [18].

## Materials and methods

### Reagents and chemicals

All reagents and chemicals were of analytical grade and applied without further purification. HPLC-grade deionized water was used during all experiments.

α-KG, 5-HMF, NASeLM, NALM, and glucose were supplied by CYL-Pharma (Lassnitzhöhe, Austria). NaCl, KCl, KH_2_PO_4_, MgSO_4_, CaCl_2_, NaHCO_3_, and dextrose were obtained from Roth (Karlsruhe, Germany). Acetonitrile, ammonium acetate, 1-butanol, ethanol, HPLC-grade water, hydrogen peroxide, and HCl were obtained from Merck (Darmstadt, Germany). Albumin from human serum, angiotensin 1-7 acetate salt hydrate, butylated hydroxytoluene (BHT), 2,4-dinitrophenylhydrazine (DNPH), ethyl acetate, guanidine-hydrochloride, malondialdehyde tetra-butyl-ammonium salt (MDA), 2-thiobarbituric acid (TBA), trichloroacetic acid (TCA), and tris(hydroxymethyl)aminomethane (Tris) were obtained from Sigma-Aldrich (Vienna, Austria).

### Animals

Three-month-old male Wistar rats, with a weight between 300 and 450 g, were obtained from Medical University of Vienna (Department of Biomedical Research). The animals were kept according to the Austrian animal welfare act. They were housed in a room with a temperature between 20 and 24 °C, 55% ± 10% relative humidity, and a 12 h light/12 h dark cycle. Animal experiments were conducted in compliance with the Austrian law on experimentation with laboratory animals. The health of the animals was periodically checked and high hygienic standards were strictly observed. Chicken hearts were obtained from a slaughterhouse.

### Apparatus

During the warm ischemia experiments on rat hearts, an IH-5 Langendorff device from Harvard Apparatus and the HSE-isoheart software were applied.

For both rat and chicken hearts, HPLC analysis was carried out with an Agilent Technologies 1260 Infinity Quat Pump VL and a Merck-Hitachi LaChrom L-7400 UV-detector. A LiChroCart^®^ RP-18 endcapped (5 µm) column (250-4) and a mobile phase containing 100 mM ammonium acetate buffer (100 mM) and acetonitrile (50 + 50 (v/v)) were applied with a flow of 1 mL/min. Samples were injected via a 100-µL loop and detected at a wavelength of 370 nm or 532 nm. Peak areas were evaluated with the Agilent ChemStation Software.

### Preparation of applied solutions

For all experiments, Krebs–Ringer solution (KRS) was used as control solution (negative control). Krebs–Ringer stock solution was composed of 2.37 M NaCl, 0.094 M KCl, 0.024 M KH_2_PO_4_, 0.023 M MgSO_4_, and 0.05 M CaCl_2_. 100 mL of this stock solution, 0.05 M NaHCO_3_, 0.023 M dextrose, and water ad 2 L were mixed to get KRS.

To obtain the multi-component solution 62 mM α-KG, 24 mM 5-HMF, 0.5 mM NALM, and 0.008 mM NaSeLM as effective ingredients and 166 mM glucose were dissolved in water. The ratio of each component has been selected based on preliminary investigations with regard to pharmacological adverse effects. The pH of the final solution was adjusted to 5.7 with 0.1 M NaOH.

For Langendorff experiments, a dilution of the multi-component solution with KRS (20 + 80; v/v)—further referred to as D20—was prepared. For physiological reasons, the tested solutions (D20, KRS) contained 0.02% albumin.

For chicken heart experiments, pure multi-component solution, KRS, and dilutions of the multi-component solution with KRS at the ratios 40 + 60 and 10 + 90 were applied. All tested solutions were mixed with 10% hydrogen peroxide 1 + 1 (v/v) for oxidative stress simulation or with water 1 + 1 (v/v) as basal value. Thus, the final investigated dilutions resulted in the concentrations of 50% (D50), 20% (D20), and 5% (D5). For single ingredient experiments, solutions with just one ingredient were prepared with the same concentration of the ingredient as in the pure multi-component solution. Each substance was dissolved in water and adjusted to pH 5.7 with 0.1 M NaOH/0.1 M HCl. The solutions were mixed 1 + 1 (v/v) with 10% hydrogen peroxide or water, resulting in a final concentration as in D50.

All solutions were filtered through a 0.45-µm membrane and stored at 4 °C in the refrigerator.

### Treatment of isolated rat hearts

Rats were anaesthetized with ketamine s.c. (70–100 mg/kg). The heart was removed quickly, immediately placed in ice-cold KRS (pH 7.4), and cannulated onto the Langendorff apparatus via aorta. The retrograde perfusion pressure was maintained at 70 mmHg ± 10 mmHg. The perfusion solutions were warmed at 37 °C ± 1 °C and infused continuously with 95% O_2_ + 5% CO_2_ throughout the experiment.

**Fig. 1:**
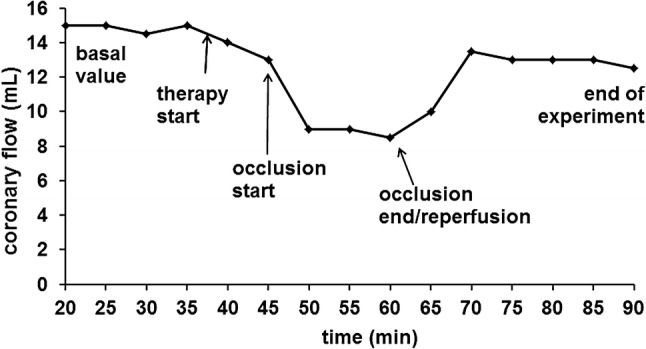
Langendorff experiment design

All hearts were purged with pure KRS during the stabilization period. After 35 min, the therapy period started by using D20 or remaining with KRS. After 10 min of therapy, warm ischemia was established for 15 min by occlusion of a coronary artery, followed by reperfusion for 30 min. The experiment was finished after 90 min. Figure 1 shows the experimental design via a typical coronary flow alteration. For non-oxidized hearts, no occlusion (= no ischemia) was established in order to get basal values for comparison.

At the end of the experiment, every single heart was divided into two parts. One part was blast-frozen with liquid nitrogen and stored at − 80 °C until investigation of oxidative stress markers. The other part was stored in formalin and used for histological and immunohistochemical analysis.

### Treatment of chicken hearts

Chicken hearts were prepared as described by Russ et al. [18]. Briefly, the hearts were sliced into 0.5 cm pieces. 2 g quantities of those were incubated in each 10 mL of oxidizing solution for 60 min. For non-oxidized values the solutions were mixed with water instead of hydrogen peroxide in order to get basal values for better comparison. Afterward the solutions were decanted and the remaining tissue was washed twice with water. Finally, the heart tissue was frozen with liquid nitrogen and pestled for homogenization. For determination of the oxidizing parameters, 100 mg quantities was mixed with 1 mL Tris buffer, vortexed, centrifuged and the supernatant was used for derivatization.

### Investigation of oxidative stress markers

Liquid nitrogen frozen tissue samples (chicken and rat) were pestled for homogenization. 100 mg of the obtained powder were mixed with 1 mL of 10 mM Tris–HCl buffer pH 7.4, centrifuged (6500 rpm, 4 °C, 30 min) and the supernatant was used for further investigation of both CPs and MDA. These experiments were carried out according to Russ et al. [18]. DNPH was applied for CP detection, TBA was used as reagent for MDA analysis. After preparation, every sample was investigated with HPLC/UV–Vis. External standards (DNPH, MDA) were daily freshly prepared and applied for accurate determination.

All experiments were carried out with 5 different hearts (*n* = 5) and determined twice. Results were calculated in nmol/g tissue. Values of hearts without occlusion/hydrogen peroxide (= without oxidative stress) were set as 100%. Values of tested hearts with occlusion/hydrogen peroxide (= exposure to oxidative stress) were compared and calculated as raise in %. For statistical significance, the *p* values were calculated as independent *T* test samples with IBM Statistical Program of Social Sciences (SPSS) statistics 25 software. A *p* value < 0.05 was considered as statistically significant.

### Histological and immunohistochemical analysis

After treatment of isolated rat hearts, the particular material was fixed in 10% neutrally buffered formalin and embedded in paraffin. From obtained blocks, 4-μm-thick sections were cut, stained with hematoxylin and eosin (HE), and immunohistochemically stained with antibodies against nitrotyrosine (A21285, 1:200, Thermo Fischer Scientific, Waltham, MA, USA) and 4-Hydroxynonenal (4-HNE) (ab46545, 1:200, AbCam, Cambridge, MA, USA).

Analysis of nitrotyrosine was performed using UltraView detection kit (Ventana Medical Systems, Inc., Tucson, AZ, USA) on Benchmark Ultra slide staining instrument (Ventana Medical Systems, Inc., Tucson, AZ, USA), after pre-treatment with CC1 for 80 min. 3,3’-diaminobenzidine (DAB) substrate-chromogen was used for visualization. 4-HNE was stained on DAKO Autostainer (DAKO, Glostrup, Denmark), after pre-treatment with Natrium-Citrat-Puffer pH 6.0 for 40 min at 150 Watt. EnVision Kit 5007 (DAKO, Glostrup, Denmark), and DAB were used for detection. Each immunostained section was analyzed and evaluated based on the average intensity of staining as: negative or positive (weak, intermediate or strong).

## Results

### Oxidative stress on isolated rat hearts

Hearts treated with the diluted multi-component solution D20 showed an improvement after coronary artery occlusion compared to hearts treated with KRS. Functional parameters measured at the Langendorff experiment before occlusion and at the end of reperfusion (average of last 15 min of reperfusion) are summarized in Table 1. All standard deviations were < 13%. Additionally, normal sinus rhythm in reperfusion was achieved faster when applying D20 solution (average 15 min KRS vs. 2 min D20).

**Table 1:** Functional assessment of the hearts subjected to ischemia reperfusion injury

	Heart rate (bpm)	LVDP (mmHg)	+ dP/dt_max_ (mmHg/s)	− dP/dt_max_ (mmHg/s)	Coronary flow (mL/min)
KRS					
Before occlusion	287.40	100.72	3718.47	2243.75	15.42
End of reperfusion	505.40	66.73	2973.45	1584.85	15.05
D20					
Before occlusion	269.63	102.62	3632.87	2255.50	14.46
End of reperfusion	203.66	97.19	3166.59	1829.17	15.75

**Fig. 2:**
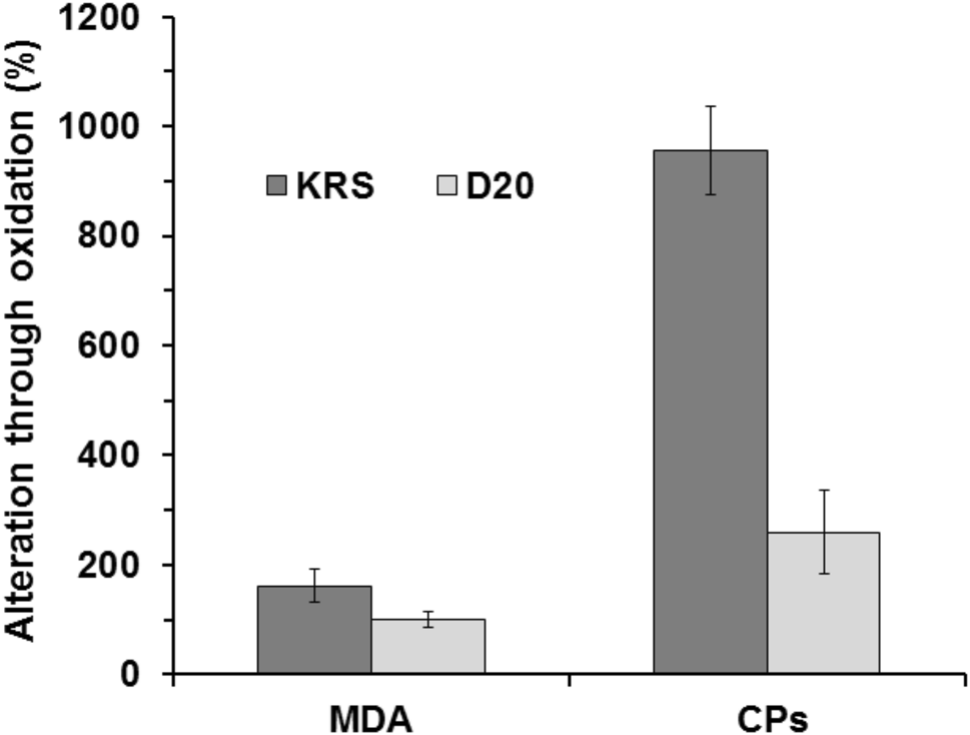
Increase of oxidative stress markers in isolated rat hearts through occlusion of a coronary artery

In order to obtain information about oxidative stress through occlusion–resulting via myocardial infarction–and its effect occurring in the differently treated hearts (KRS, D20), MDA and CPs were studied. This procedure seems to be most appropriate for real life conditions. Both parameters were examined in the heart tissue with HPLC/UV–Vis. As can be seen in Fig. 2, an increase of both parameters in hearts treated with KRS occurred. MDA showed a rise of about 160% through oxidative stress, CPs increased about 950% compared to hearts without oxidative stress. Treatment with D20 resulted in a MDA value that remained at the level of about 100%, and an increase of about 260% CPs. MDA and CPs basal values for rat hearts were 18.64 ± 0.76 nmol/g tissue and 2.16 ± 0.34 nmol/g tissue, respectively.

**Fig. 3:**
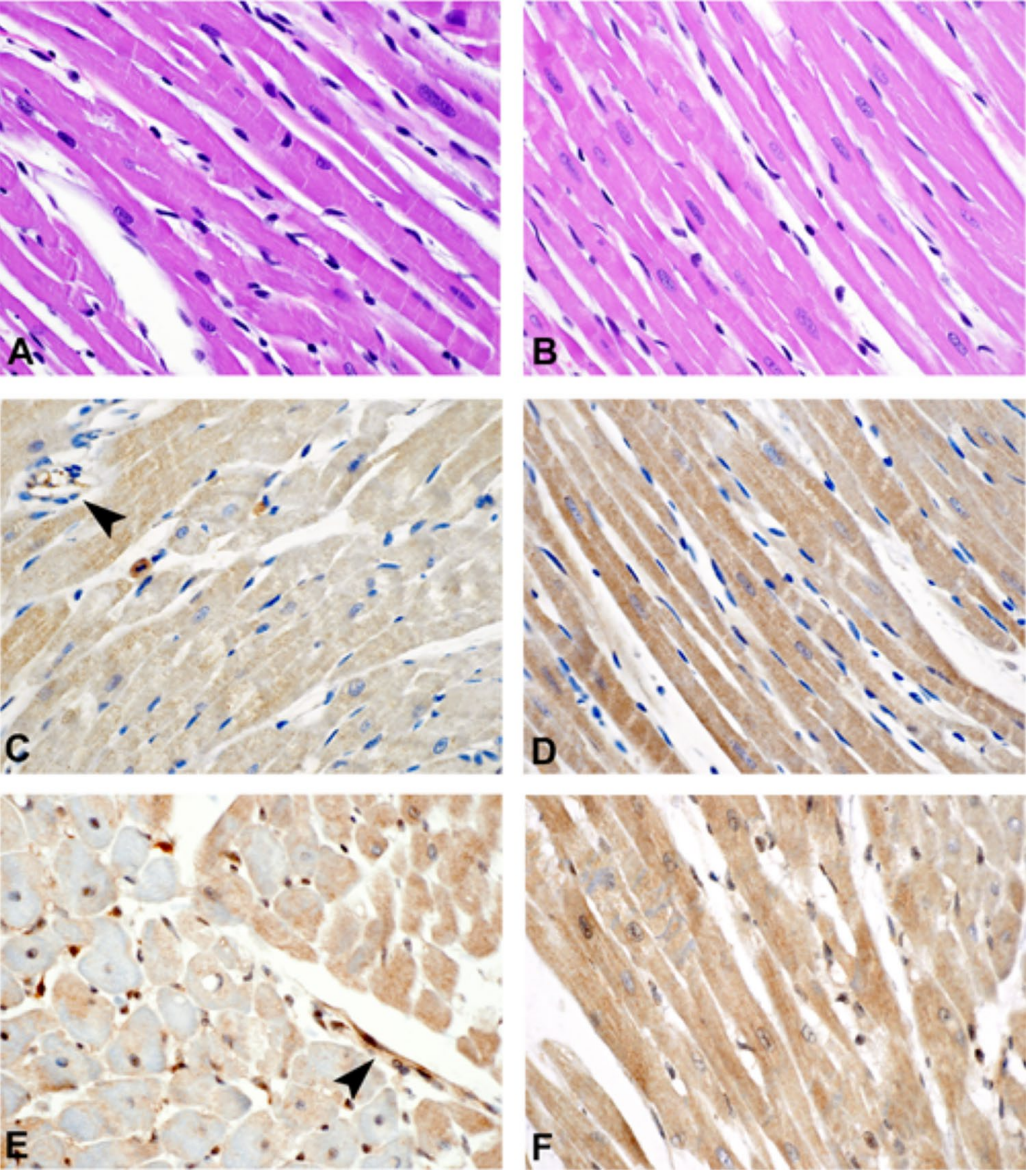
Comparison of histological and immunohistochemical findings. HE staining: D20 group (**a**) and KRS group (**b**); 4-HNE-staining: D20 group (**c**) and KRS group (**d**); nitrotyrosine staining: D20 group (**e**) and KRS group (**f**). Note positive endothelial cells in C&E (arrowhead) as positive internal control. Original magnification × 400

For statistical significance, *p* values were calculated by comparing KRS and D20. For MDA and CPs investigations, the *p* value was found to be 0.001 and 0.013, respectively. Furthermore, histological analysis of rat heart tissue treated with D20 or KRS and stained with HE showed in both cases myocytes with enlarged, focally centralized nuclei. Nevertheless, immunostainings with antibodies against 4-HNE and nitrotyrosine exhibited intermediate intensity of staining in KRS group, while the staining in D20 group was weak (Fig. 3). Note positive endothelial cells in Fig. 3c and e (arrowhead) as positive internal control.

### Oxidative stress on chicken heart tissue

**Fig. 4:**
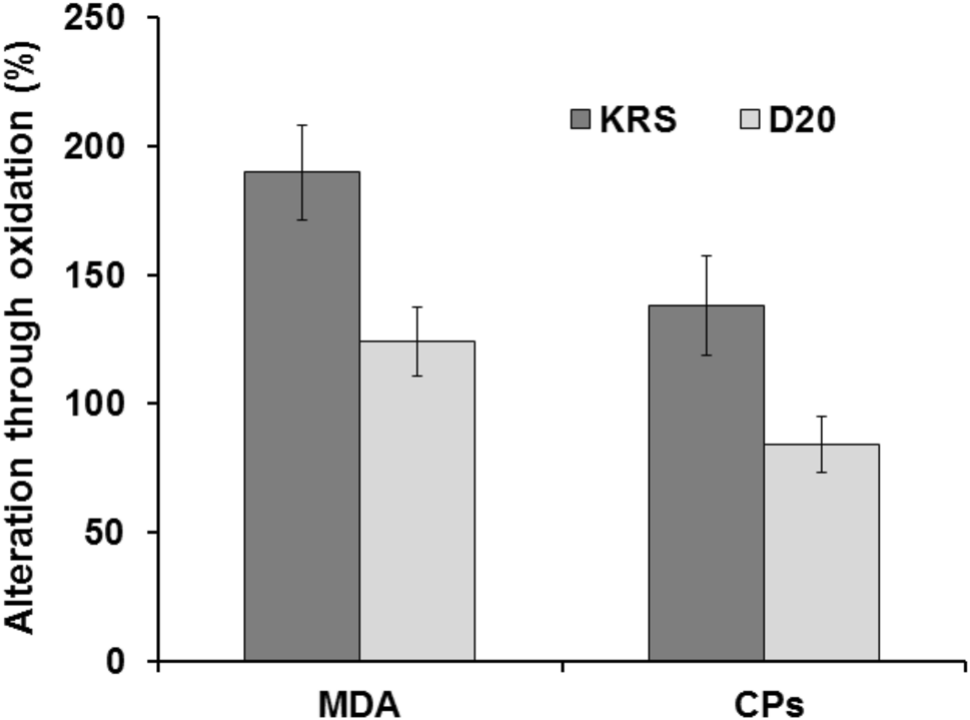
Increase of oxidative stress markers in chicken heart tissue through treatment with 5% hydrogen peroxide

**Fig. 5:**
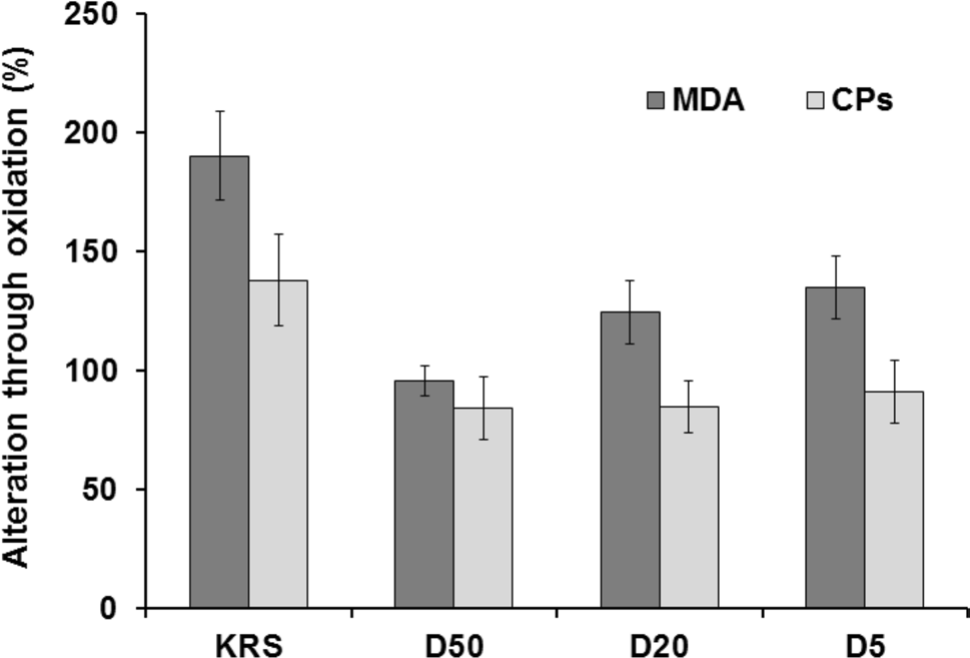
Influence of different concentration solutions and 5% hydrogen peroxide on oxidative stress markers in chicken heart tissue

**Fig. 6:**
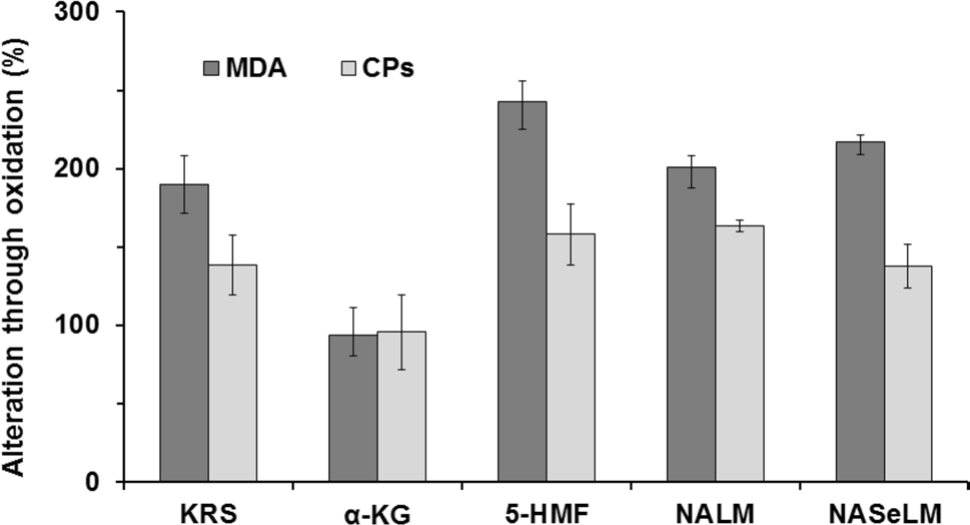
Influence of single ingredient solutions and 5% hydrogen peroxide on oxidative stress markers in chicken heart tissue

Chicken hearts were oxidized with hydrogen peroxide and also examined considering the oxidative stress parameters MDA and CPs. Figure 4 displays a rise of MDA (about 190%) and CPs (about 140%) in hearts treated with KRS compared to non-oxidized tissue (= basal value). On the other hand, treatment with D20 led to an increase of about 120% MDA and no rise in CPs. For concentration studies, dilutions of 50% (D50), 20% (D20), and 5% (D5) were examined. Figure 5 shows the outcome of these investigations. Single component studies were carried out at the same concentrations of ingredients as used in D50. Figure 6 displays the results of the single ingredient experiments. 5-HMF, NALM, and NASeLM treated hearts showed a rise of MDA between 200 and 350% and an average similar rise of 150% considering CPs. In contrast, MDA and CPs levels in hearts treated with α-KG remained the same as in hearts without additional hydrogen peroxide treatment (= without oxidative stress). The relevance of the performed studies can be seen from the statistical data given in Table 2.

**Table 2:** Statistical data of investigations in chicken heart tissue

	MDA nmol/g tissue	*p* value (compared to KRS)	CP nmol/g tissue	*p* value (compared to KRS)
Basal value	47.45 ± 2.85		1.83 ± 0.30	
KRS	90.08 ± 16.76		2.53 ± 0.48	
D20	58.96 ± 7.81	0.015	1.54 ± 0.17	0.009
D50	45.24 ± 2.91	0.002	1.54 ± 0.20	0.009
D5	63.94 ± 8.51	0.032	1.66 ± 0.22	0.016
α-KG	44.42 ± 7.97	0.003	1.75 ± 0.42	0.049
5-HMF	115.28 ± 15.35	0.068	2.89 ± 0.56	0.357
NALM	95.31 ± 7.31	0.588	2.99 ± 0.11	0.112
NASeLM	102.85 ± 5.09	0.195	2.52 ± 0.35	0.986

## Discussion

Herein, a supposed antioxidative effect of a multi-component solution against ROS occurring through oxidative stress in heart tissue was examined via MDA and CPs determination. The parameters MDA and CPs cover the field of oxidative protein and lipid damage and therefore provide meaningful information on cell decay of cardiomyocytes. Oxidative stress was induced either through myocardial infarction or through application of hydrogen peroxide.

Isolated rat hearts were examined on a Langendorff device. The therapeutic approach of D20 was applied for this study because of former experiments [13]. All Langendorff observations indicate a positive influence of D20 during ischemia/oxidative stress. This effect was also confirmed in immunostaining experiments by means of detecting 4-HNE and nitrotyrosine. Considering the investigated oxidative stress parameters, D20 reduced MDA and CPs in these experiments with a significance of *p* < 0.05 compared to a negative control. The same trend was found in chicken heart tissue studies.

For further analysis, commercially available chicken heart tissue was used in order to avoid material directly from living animals for the described purpose. Experiments were carried out regarding concentration and single components of the tested solution.

At concentration studies, MDA levels were lower at all concentrations compared to the negative control with KRS. The effect seems to be slightly concentration dependent with a result of no rise with D50 treated hearts, and a rise of about 120% and 130% for D20 and D5 treated hearts. Considering CPs, all concentrations revealed a similar result with no rise compared to non-oxidized tissue.

Single component studies were carried out at the same concentrations of ingredients as used in D50 because of the best influence of this concentration as given above. The results of these experiments identify α-KG as effective antioxidative ingredient of the tested solution in chicken heart tissue which is treated with hydrogen peroxide. All other ingredients seem to have less/no antioxidative effect in these experiments. Although 5-HMF is reported to have antioxidative effects [7, 8, 19] even in liver tissue [20, 21], this failed to proof under oxidizing conditions in chicken heart tissue. α-KG is known for its antioxidative effect for example in fruit flies [22, 23]. According to the review of Zdzisinksa et al., it inhibited oxidative stress through ammonium acetate and ethanol in vivo in rats and showed positive antioxidative effects during hepatocarcinogenesis and cataract formation [6]. In the body it plays a role in the citrate cycle and is reported to react directly with hydrogen peroxide, forming carboxylic acid, CO_2_, and H_2_O [5, 6]. However, it has never been studied considering this effect in heart tissue exposed to oxidative stress before.

Overall, the results of this study showed that the multi-component solution exhibited an antioxidative effect against the induced oxidative stress. Both investigated parameters were increased through oxidative stress in the control group, but stayed at basal level or were less increased through application of the multi-component solution. Additionally, α-KG was identified as antioxidative component in chicken heart tissue in these experiments. Nevertheless, metabolic processes might influence the effectivity of the different ingredients in vivo. Therefore, all ingredients of the multi-component solution (single and together) may exhibit a positive effect against oxidative stress. In order to identify these possible effects, in vivo studies are planned.

Up to now, only few solutions are available for organ transplantations. The approach described in this study is novel and seems to be a powerful option to be transferred for heart transplantations in order to improve shelf life and avoid graft failure.
